# Acquired Acrodermatitis Enteropathica: A Case Study

**DOI:** 10.7759/cureus.1667

**Published:** 2017-09-08

**Authors:** Steven Kelly, John W Stelzer, Nathan Esplin, Ahsan Farooq, Olga Karasik

**Affiliations:** 1 Medical Student, University of Central Florida College of Medicine; 2 University of Central Florida College of Medicine; 3 Internal Medicine, University of Central Florida College of Medicine

**Keywords:** zinc-deficiency, acquired acrodermatitis enteropathica, rash, dermatitis

## Abstract

We present a case of severe acquired acrodermatitis enteropathica in a vegan adult female with multiple underlying comorbidities. Acquired acrodermatitis enteropathica or zinc-deficiency dermatitis is the most common diagnosis than many practitioners realize with up to 10% of the patients in developed nations with the risk of zinc deficiency. The condition can be difficult to diagnose due to many similarly-presenting conditions. Furthermore, comorbid conditions in the patients can serve as confounders to the diagnosis. The symptoms are often extremely distressing for the patients, though the treatment is simple and clinical improvement occurs rapidly with appropriate care. We recommend a high index of suspicion to practitioners as well as a low-threshold for initiating treatment in the patients with any clinical symptoms of the condition.

## Introduction

Intractable rash in a patient with multiple comorbidities can be a complex medical challenge. This describes a case of zinc deficiency dermatitis that developed secondary to pancreatic insufficiency, a vegan diet with no supplementation and malnutrition in the patient with bronchiectasis, Mycoplasma pneumoniae, transaminitis and hemolytic anemia.

## Case presentation

A 43-year-old female presented to the emergency department with a nearly full body rash, including the vaginal introitus and perianal skin. The patient described the rash as “blistered and flaky.” She also had peripheral edema, glossitis, cheilitis, and alopecia described as “my hair has been falling out in chunks.” She reported a loss of appetite from the glossitis along with increasing diarrhea and abdominal pain. She further stated that she had recent onset of decreased focus and memory. The patient presented two weeks prior with a similar rash that had been less widespread. She reported that the rash started on her feet and mouth before spreading to her knees and was accompanied by lower extremity swelling, cheilitis, and glossitis. At that time, she was presumed to have scabies and also tested positive for Staphylococcus in a skin sample. She was treated with vancomycin before leaving the hospital against medical advice prior to the completion of workup and treatment. She now returned, two weeks later, with an acute worsening of the rash accompanied by worsening memory and concentration difficulties.

The patient had a lifetime history of multiple allergies, food intolerances/sensitivities, asthma, abdominal pain and diarrhea, and rash due to certain foods including dairy products. She stated she had been diagnosed with a liver disease several months before the admission and that she had been taking more than 4000 mg Acetaminophen per day in the months leading up to the diagnosis. It was also noted in several past medical records that this patient had a lengthy history of alcohol abuse. She was also admitted due to anxiety, depression, insomnia and a history of bulimia. She was admitted for an unknown amount of time seven years ago with her first attack of pancreatitis; approximately four years later, she was admitted again for several weeks with an attack of pancreatitis, during which she developed erythema, swelling, and blistering of the dorsal aspects of her feet. Other possible admissions for pancreatitis at regional hospitals were surmised from her history but were unable to be verified. She stated that she had been adherent to a vegan diet for the last 17 years to avoid rashes and diarrhea that accompanied several types of food and in the last few years, she had been unable to afford or acquire good vitamin supplementation. The patient had a history of non-compliance with her pancreatic enzyme replacement regimen due to socioeconomic factors.

On initial workup, the patient had a generalized maculopapular rash with a scale that blanched by pressure. She stated that the rash started on her dorsal feet and mouth, and then spread up her legs, genitals and perirectal areas, and distal upper extremities. The rash spared the palms, soles, and face with the exception of glossitis and cheilitis, which were present. Notable labs obtained during the time as an inpatient can be seen in Table 1.

**Table 1:** Table representing the patient lab tests.

Lab Test	Value/Result
White blood cells	13.32 billion cells/liter (normal: 3.5-10.5)
Procalcitonin	0.12 ng/mL (normal: 0.15-0.2)
Lactate	5.18 mmol/L (normal: 0.5-1.0)
Potassium	2.9 mEq/L (normal 3.5-5.0)
Erythrocyte sedimentation rate:	42 mm/hr (normal: 0-29)
Haptoglobin:	22.1 mg/dL (normal: 30-200)
Prothrombin time:	17.8 s (normal: 11-13.5)
International normalized ratio:	1.8 (normal: 0.8-1.1)
Zinc:	30 µmol/L (normal: 70-100)
Mycoplasma pneumoniae Immunogobulin M (IgM):	positive
Stool occult blood:	negative
Cryoglobulin:	none detected
Cold agglutinin titer:	negative
Anti-Nuclear antibody pattern:	negative
Heparin-induced antibody:	negative

A biopsy of an area of rash from the patient’s left leg was performed and analysis revealed the following: epidermal ulcer, spongiosis, dermal fibrosis and mixed inflammation including many dermal eosinophils. Immunoglobulin G (IgG): negative, Immunoglobulin M (IgM): negative, Immunoglobulin A (IgA): negative, C3: negative, fibrinogen: negative.

Clinical course

The patient was treated for Mycoplasma pneumoniae with antibiotics and breathing treatments. Pancreatic enzymes were provided and steroid cream was given for symptomatic treatment of the rash. Dermatology was consulted, who ultimately determined that the skin findings represented zinc deficiency dermatitis and recommended oral zinc therapy. The rash demonstrated significant improvement over the next two days and was mostly clear by the third day of zinc treatment. The patient also reported improvements in her confusion and could remember details of her past medical history that she previously was unable to provide. Her hospital course was complicated by hemolysis of unknown etiology. This was possibly related to extrapulmonary Mycoplasma pneumoniae, which was treated with infusion of packed red blood cells and prednisone. Prior to discharge, the patient provided informed consent for the case write-up on her condition and clinical course.

## Discussion

Zinc is concentrated in the epithelium and plays several key roles in that area. Those roles include supporting structural integrity, cellular survival, wound healing, and anti-inflammatory function [1].

It is found in many different dietary items but is particularly concentrated in animal-sourced foods. Our bodies have no clear storage mechanism for zinc, and it is thus important to receive small amounts of it regularly in the diet [2]. Worldwide, an estimated 17.3% of the population is at risk for zinc deficiency [3]. However, as is often the case with diseases resulting from nutritional deficiencies, there is a disparity between developing and developed nations, with deficiency being most common in Sub-Saharan Africa and South Asia [3]. The cases of zinc deficiency in developed nations, such as the United States, are less common but have been increasing over the last 20 years, currently estimated to occur in 3-10% of the population [4].

Zinc deficiency dermatitis is described as either “acrodermatitis enteropathica” or “acquired acrodermatitis enteropathica,” depending on the specific etiology. Though the treatments for the two disease states are essentially the same, they have distinct etiologies. Acrodermatitis enteropathica refers specifically to a zinc deficiency with a genetic basis resulting in the loss of the zinc transporter [5] and presents in infancy. Acquired acrodermatitis enteropathica refers to dietary zinc deficiency. It may also present in infants, especially in the premature, due to their greater physiologic demand for zinc [6]. However, it most often occurs in acquired states of nutritional deficit such as anorexia nervosa [7], celiac disease [8], and other conditions of malnutrition.

Regardless of the specific etiology, zinc deficiency typically presents with erythematous, desquamative, and erosive acral, periorificial, and distal extremity dermatitis, skin lesions, cheilitis, stomatitis, alopecia, and diarrhea [7]. Clues to the mechanism of injury in zinc deficiency are found in the characteristic pattern of signs and symptoms. The symptoms represent a contact dermatitis that develops after normal contact with sweat, feces, and other irritants, due to poor defense and inadequate healing response. This is intuitive when considered in the context of the well-established role of zinc in immune function and healing [1]. Furthermore, in addition to the characteristic skin findings, patients with severe/chronic zinc-deficiency dermatitis can also develop mental status changes. Most commonly it is associated with depression, and treatment with zinc for depression has demonstrated significant efficacy [9]. Our patient suffered from long-term depression and anxiety and had more recently developed confusion and memory loss. Zinc supplementation was effective for resolving the latter symptoms within days of initiating treatment.

Diagnosis of zinc deficiency dermatitis can be challenging in an adult due to a myriad of confounders, as the lesions can mimic a number of other disorders (Table 2). Serum zinc levels can drop in the presence of inflammation, and plasma zinc only represents 0.1% of total body zinc. Therefore, zinc levels in the plasma may not represent the total body zinc balance [10]. The providers should have a high index of suspicion for the patients presenting with the characteristic rash, and a low-threshold for implementing zinc supplementation. Treatment with zinc supplementation confirms the diagnosis when rapid improvement of symptoms is observed [10].

**Table 2:** Table representing the differential diagnosis for acquired acrodermatitis enteropathica. (+): Feature from this patient's presentation/work-up that supports the listed disease or syndrome. (-): Feature from this patient's presentation/work-up that does not support the listed disease or syndrome.

Condition	Supporting and Opposing Features
Acquired Acrodermatitis Enteropathica	(+) The rash was accompanied by cheilitis, stomatitis, and alopecia
	(+) Rash cleared significantly within two days of receiving zinc supplementation
	(+) Characteristic symptoms of zinc deficiency did not develop until a mature age
Acrodermatitis Enteropathica	(+) The rash was accompanied by cheilitis, stomatitis, and alopecia
	(+) Rash cleared significantly within two days of receiving zinc supplementation
	(˗) Characteristic symptoms of zinc deficiency did not develop until a mature age
Extrapulmonary manifestation Mycoplasma pneumoniae	(+) Positive Mycoplasma pneumonia IgM
	(+) Can present with skin findings and hemolysis
	(˗) Cold agglutinins negative in this patient
Drug reaction with eosinophilia and systemic symptoms (DRESS) syndrome	(+) Skin biopsy demonstrated dermal eosinophils
	(+) Rash spread and worsened after one dose of vancomycin on the first visit
	(˗) Rash was present before beginning medical therapy
Pancreatitis	(+) Patient has history of pancreatitis that twice co-occurred with a rash that resolved with treatment of pancreatitis
	(˗) No characteristic signs and symptoms of pancreatitis
Lupus	(+) Can present with rash and neurologic symptoms
	(˗) Patient had a negative antinuclear antibody* (*ANA) titer
Inflammatory bowel disease	(+) Lifetime history of abdominal pain
	(+) Nutritional deficiencies present
	(˗) Negative Fecal Occult Blood Test
Cryoglobulinemia	(+) Skin, Neurologic, and gastrointestinal (GI) signs and symptoms often present
	(+) The patient gave the history of unknown “liver disease” diagnosed months before admission.
	(˗) Negative Cryoglobulin testing

## Conclusions

In conclusion, this case demonstrates the necessity of considering zinc deficiency in the differential diagnosis of any patient who presents with a constellation of rash, diarrhea, alopecia, and mood or mental status changes. The potential difficulty of the diagnosis due to multiple underlying medical conditions is well demonstrated in this case. Additionally, this case demonstrates several other conditions that should be taken into consideration during the workup of a patient with suspected acquired acrodermatitis enteropathica. We recommend a low threshold for implementing zinc supplementation in those who present with the characteristic findings.
